# Salidroside alleviates hepatic ischemia–reperfusion injury during liver transplant in rat through regulating TLR-4/NF-κB/NLRP3 inflammatory pathway

**DOI:** 10.1038/s41598-022-18369-4

**Published:** 2022-08-17

**Authors:** Yanyao Liu, Zilun Lei, Hao Chai, Quan Kang, Xiaoyan Qin

**Affiliations:** 1 Department of General Surgery and Trauma Surgery, Children’s Hospital of Chongqing Medical University, Ministry of Education Key Laboratory of Child Development and Disorders, National Clinical Research Center for Child Health and Disorders, China International Science and Technology Cooperation Base of Child Development and Critical Disorders, Chongqing Key Laboratory of Pediatrics, Chongqing, China; 2 Department of Hepatobiliary Surgery, The First Affiliated Hospital of Chongqing Medical University, Chongqing, China

**Keywords:** Liver diseases, Liver

## Abstract

Salidroside has anti-inflammatory, antioxidant and hepatoprotective properties. However, its effect on hepatic ischemia–reperfusion injury (IRI), an unavoidable side effect associated with liver transplantation, remains undefined. Here, we aimed to determine whether salidroside alleviates hepatic IRI and elucidate its potential mechanisms. We used both in vivo and in vitro assays to assess the effect and mechanisms of salidroside on hepatic IRI. Hepatic IRI rat models were pretreated with salidroside (5, 10 or 20 mg/kg/day) for 7 days following liver transplantation while hypoxia/reoxygenation (H/R) model of RAW 264.7 macrophages were pretreated with salidroside (1, 10 or 50 μM). The effect of salidroside on hepatic IRI was assessed using hematoxylin–eosin staining, terminal deoxynucleotidyl transferase dUTP nick-end labeling staining, qRT-PCR, immunosorbent assay and western blotting. Our in vivo assays showed that salidroside significantly reduced pathological liver damage, serum aminotransferase levels and serum levels of IL-1, IL-18 and TNF-α. Besides, salidroside reduced the expression of TLR-4/NF-κB/NLRP3 inflammatory pathway associated proteins (TLR-4, MyD88, p-IKKα, p-IKKβ, p-IKK, p-IκBα, p-P65, NLRP3, ASC, Cleaved caspase-1, IL-1β, IL-18, TNF-α and IL-6) in rats after liver transplantation. On the other hand, data from the in vitro analysis demonstrated that salidroside blocks expression of TLR-4/NF-κB/NLRP3 inflammatory pathway related proteins in the RAW264.7 cells treated with H/R. The salidroside-specific anti-inflammatory effects were partially inhibited by the TLR-4 agonist lipopolysaccharide. Taken together, our study showed that salidroside inhibits hepatic IRI following liver transplantation by modulating the TLR-4/NF-κB/NLRP3 inflammatory pathway.

## Introduction

Hepatic ischemia/reperfusion injury (IRI) is an unavoidable pathological event in many liver surgical procedures, especially liver transplantation^1,2^. Hepatic ischemia can cause metabolic problems and an inflammatory microenvironment in the liver, which could lead to graft rejection, hepatic pathological injury or apoptosis and early graft dysfunction^3,4^. The underlying pathogenesis of hepatic IRI is complicated, thus posing many barriers to early detection, diagnosis and treatment.

Previous studies have shown that innate immune system plays an important role in hepatic IRI, acting as the first line of defense against infection or injury by activating pattern recognition receptors (PRRs) such as Toll-like receptors (TLRs) as well as nucleotide-binding oligomerization domain-like receptors (NLRs)^5,6^. Primary TLRs expressed in hepatocytes and macrophages include TLR-2, TLR-3, TLR-4 and TLR-9 of which TLR-4 is involved in inflammatory responses caused by hepatic IRI^7^. TLR-4 signaling promotes MyD88-dependent pathways, which leads to the activation of nuclear factor kappa B (NF-κB), which is essential for production of proinflammatory cytokines^8,9^. Induction of the signaling pathway of NF-κB is one of the main mediators of inflammation and defines the development and progression of hepatic IRI^10,11^. As the most studied tissue-damage detector, the NLR family pyrin domain-containing 3 (NLRP3) inflammasome is composed of NLRP3, apoptosis-associated speck-like protein (ASC) and caspase-1, which is prominent in initiating and propagating the inflammatory responses^12^. Previous evidence suggests that the NF-κB signaling leads to NLRP3 activation that participates in the pathogenesis of hepatic IRI^13^. Thus, the TLR-4/NF-κB/NLRP3 inflammatory pathway might be involved in hepatic IRI.

Salidroside, a compound extracted from various Rhodiola plants, is widely used in treatment of ischemia stroke, Alzheimer’s disease, and cardiovascular diseases^14,15^. Recent data has shown that salidroside exerts a wide range of pharmacological activities, such as anti-hypoxia, anti-inflammation and antioxidant activity^16,17^. Furthermore, several studies have reported that salidroside could alleviate liver inflammation in furan-induced animals by modulating the NF-κB/NLRP3 signaling pathway, and plays a unique protective role in hepatic IRI^18^. In this study, we investigated the potential of salidroside to alleviate hepatic IRI and its underlying mechanisms using orthotopic liver transplant rat models.

## Materials and methods

### Ethical statement

All experiments were approved and performed in accordance with relevant institutional licensing committee guidelines and regulations (Ethics committee of Animal and Human Experimentation of Chongqing Medical University). The study was carried out in compliance with the ARRIVE guidelines. Euthanasia of animals is by CO2 exposure followed by cervical dislocation in compliance with the AVMA guidelines for the euthanasia of animals (2020).

### Animals and orthotopic liver transplantation models

Chongqing Medical University's experimental animal center provided inbred male Sprague–Dawley rats (SD) (SPF grade, 220–250 g). The animals were kept in a pathogen-free environment. The rats were randomly divided into five groups, each with twelve animals (n = 12). We employed a unique magnetic anastomosis technique to perform orthotopic liver transplantation as previously described^19^. The sham group underwent abdominal incision to expose hepatic portal vein. After liver transplant, the rats in the ischemia reperfusion (I/R) group received no therapy. On the other hand, the rats in the I/R + Sal (5 mg/kg) group received salidroside (dissolved in 0.9 percent saline) (yuanyeBio, Shanghai, China) intraperitoneally for 7 days (5 mg/kg/day) before the surgery. The rats in the I/R + Sal (10 mg/kg) group were given salidroside (dissolved in 0.9 percent saline) intraperitoneally for 7 days (10 mg/kg/day) before the surgery. In addition, the rats in the I/R + Sal (20 mg/kg) group received salidroside (dissolved in 0.9 percent saline) intraperitoneally for 7 days (20 mg/kg/day) before the surgery. After orthotopic liver transplantation, the animals were euthanized at different times, and then liver tissues and sera were collected and frozen at − 80 °C.

### In vitro study design and cell model

The RAW 264.7 cells were obtained from the Chinese Academy of Sciences' Type Culture Collection's Cell Bank and grown at 37 °C and 5% CO_2_ in F12/DMEM (Gibico Grand Island, USA) with 10% fetal bovine serum (Gibco, Australia). A hypoxia/reoxygenation (H/R) cell model was used to simulate hepatic IRI. Briefly, RAW 264.7 macrophages were placed in a tri-gas incubator (Thermo, MA, USA) with a 1% O_2_ and 5% CO_2_ condition. The cells were taken for additional tests after 6 h of hypoxia followed by 6 h of reoxygenation. With or without lipopolysaccharide (LPS) (25 mol/L, Beyotime, Shanghai, China), the cells were treated with 1, 10, or 50 M salidroside. Control cells were cultured in F12/DMEM (Gibico Grand Island, USA) containing 10% fetal bovine serum (Gibco, Australia) at 37 °C and 5% CO_2_.

### Aminotransferase assay

Liver enzyme kits (Jiancheng Bioengineering Institute, China) were used to measure serum aminotransferase (AST) and aspartate aminotransferase (AST) levels, according to the manufacturer's instructions.

### Determination of cytokines

Enzyme-linked immunosorbent assay (ELISA) kits were used to measure IL-1, IL-18, and tumor necrosis factor-α (TNF-α) in the cell culture supernatants and rat sera (Neobioscience, Beijing, China), following the manufacturers’ instructions.

### Histology and TUNEL analysis

The grafts were fixed with 4% paraformaldehyde for 24 h. After being embedded, the tissues were sectioned at 5 µM and stained with HE. Suzuki scores were used to evaluate liver damage. The level of hepatic apoptosis was determined using a TUNEL kit (Beyotime, Shanghai, China), according to the manufacturer's protocol. An optical microscope (Lecia, Wetzlar, Germany) was used to analyze and image the tissues at different magnifications.

### Western blot

Total protein from the liver tissues of rats following liver transplantation and RAW 264.7 cells from the Chinese Academy of Sciences' Type Culture Collection's Cell Bank was extracted by lysis in radio immunoprecipitation assay buffer with a proteinase inhibitor cocktail. The proteins were resolved in 10% SDS-PAGE gels before transfer into polyvinylidene fluoride membranes and then blocked for 15 min with blocking buffer. The membranes were treated overnight at 4 °C with primary antibodies (Table 1), washed and then incubated with secondary antibody for 1 h at room temperature. A gel imaging system was used to detect signals generated by chemiluminescence (ChemiScope 2850, Clinx Science, Shanghai, China). We then quantified the bands using ImageJ program.

**Table 1:** Antibodies for immunofluorescence staining and western blot.

Primary antibody	Dilution concentration	Supplier
TLR-4	WB (1/300)	Abcam
MyD88	WB (1/1000)	Abcam
p-IKKα	WB (1/1000)	Beyotime
IKKα	WB (1/1000)	Beyotime
p-IKKβ	WB (1/1000)	Beyotime
IKKβ	WB (1/1000)	Beyotime
p-IKK	WB (1/1000)	Beyotime
IKK	WB (1/1000)	Beyotime
p- IκBα	WB (1/2500)	Santa Cruz
IκBα	WB (1/2500)	Santa Cruz
p-P65	WB (1/2500)	Beyotime
P65	WB (1/1000)	Beyotime
NLRP3	WB (1/1000)	Bioss
ASC	WB (1/1000)	Santa Cruz
Cleaved caspase-1	WB (1/1000)	CST
Caspase-1	WB (1/1000)	CST
IL-1β	WB (1/1000)	Beyotime
IL-18	WB (1/1000)	Beyotime
TNF-α	WB (1/1000)	Beyotime
IL-6	WB (1/1000)	Beyotime
β-actin	WB (1/1000)	Beyotime

*WB* western blotting.

### Quantitative real-time PCR (qRT-PCR)

Total RNA was extracted by TRIzol (Takara, Tokyo, Japan) according to the manufacturer's instructions, and then PrimeScript RT Reagent (Takara, Tokyo, Japan) was used to synthesize complementary DNA (cDNA). The PCR primers were provided by Sangon Biotech (Sangon Biotech, Shanghai, China), (Table 2). We used the 2^−ΔΔCt^ method to evaluate all qRT-PCR samples, which were conducted in triplicate.

**Table 2:** Primer sequences used for PCR analyses.

Name	Forward primer	Reverse primer
TNF-α	CTACGTGCTCCTCACCCACACCGT	ACCTCAGCGCTGAGCAGGTCCCCC
IL-1β	AGGGCTGCTTCCAAACCTTTGACC	ACTGCCTGCCTGAAGCTCTTGTTG
IL-6	CTGATTGTATGAACAGCGATGATG	AACTCCAGAAGACCAGAGCAGATT
IL-18	GCAGTAATACGGAGCATAAA	ATCCTTCACAGATAGGGTCA
Ly6G	TGTGCAGAAAGAGCTCAGGGGCTGG	AGTGGGGCAGATGGGAAGGCAGAGA
CD11b	TGATTGATGGCTCCGGTAG	GGACTGTGGTTTGTTGAAGGC
GADPH	CTCACTCAAGATTGTCAGCA	GTCTTCTGGGTGGCAGTGAT

### Statistical analysis

The data was reported as the mean ± SD of at least three independent replicates. For comparisons involving means of two groups, the Student's t-test was used, while one-way ANOVA was utilized for comparisons of more than two groups. GraphPad Prism version 8.0 was used to conduct the data analyses. A p < 0.05 was used to indicate statistical significance.

## Results

### Salidroside enhances liver function and reduces inflammatory cytokines in rats after liver transplant

**Figure 1:**
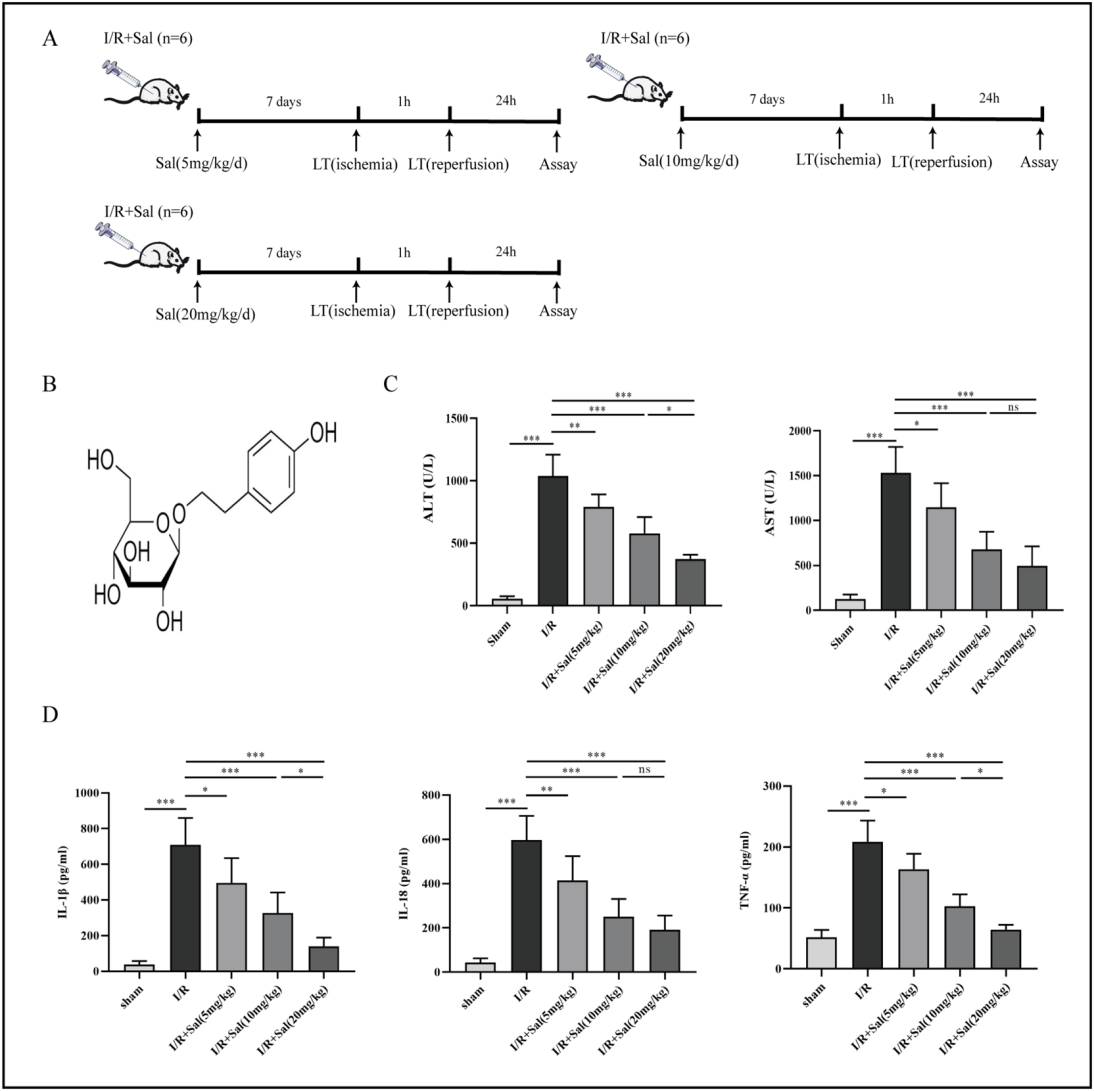
Salidroside improves liver function and decrease inflammatory cytokines in rats after liver transplantation. (**A**) Schematic diagram describing the grouping of the rats. (**B**) Chemical structure of salidroside. (**C**) Serum levels of ALT and AST in different groups. (**D**) Serum levels of inflammatory cytokines in different groups. The statistical differences among groups were assessed by one-way ANOVA. ^ns^*P* > 0.05, *P < 0.05, **P < 0.01, ***P < 0.001.

The rats were divided into three groups and received liver transplantation after salidroside intraperitoneally for 7 days (Fig. 1A). The chemical formula of salidroside is shown (Fig. 1B). Serum levels of AST and ALT are the main indicators in evaluating liver function. Our analysis showed that ALT and AST levels were significantly elevated after liver transplant. Pretreatment with increasing dosages of salidroside (5, 10, or 20 mg/kg) for 7 days decreased ALT and AST levels compared to the I/R group, demonstrating that salidroside treatment improves the recipients' liver functioning (Fig. 1C). The IL-1β, IL-18, and TNF-α serum levels were markedly higher in the I/R group compared to the control group. In comparison to the I/R group, salidroside pretreatment (5, 10 or 20 mg/kg) gradually suppressed the elevated levels of the cytokines (Fig. 1D).

### Salidroside decreases histopathological changes in rats after liver transplant

**Figure 2:**
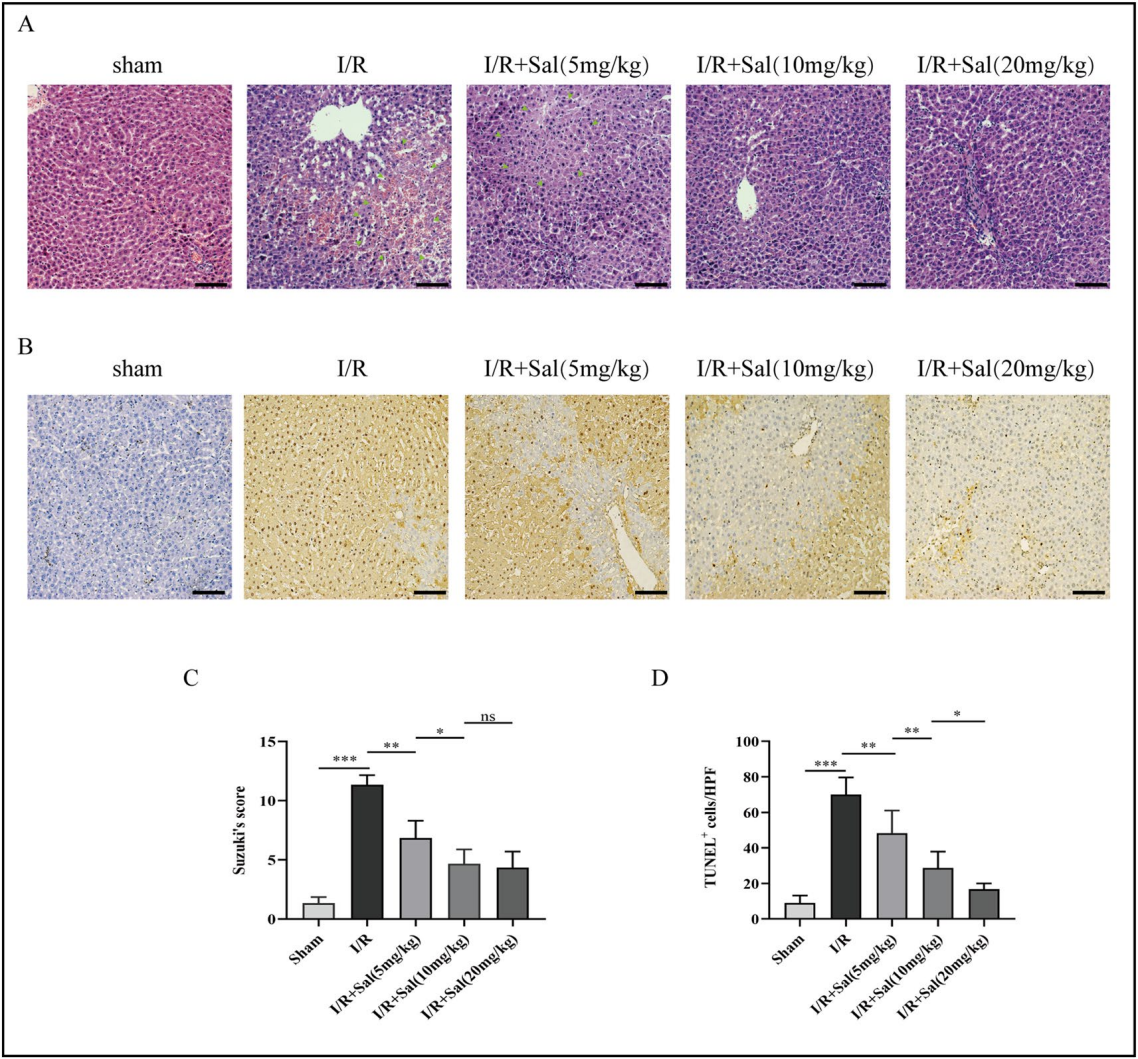
Salidroside attenuates hepatic histopathological changes and hepatocyte apoptosis of the rats following liver transplantation. (**A**) Representative HE staining images showing hepatic histological alterations (indicated with arrowheads) in the rat liver (magnification, 200; scale bar, 200 m; n = 6). (**B**) Hepatic apoptosis detected by TUNEL assay (magnification, × 200; scale bar, 200 μm; n = 6). (**C**) Hepatic IRI grading based on suzuki’s grade. (**D**) TUNEL^+^ cells of the liver based on TUNEL assay. The statistical differences among groups were assessed by one-way ANOVA. ^ns^*P* > 0.05, *P < 0.05, **P < 0.01, ***P < 0.001.

To assess the function of salidroside in IRI-induced hepatocellular damage, HE staining was used examine the severity of histopathological changes in the liver from the rats in different groups. The HE staining data showed that the degree of coagulation necrosis, architectural anomalies, and hepatocyte vacuolization were milder in the I/R + Sal (5, 10 or 20 mg/kg) group. These findings showed that salidroside pretreatment significantly reduces IRI-induced hepatic pathological changes (Fig. 2A,C).

### Salidroside attenuates hepatocyte apoptosis in rats following liver transplant

To investigate whether salidroside prevents hepatocyte apoptosis in vivo, the levels of hepatocyte apoptosis were determined using a TUNEL assay. The data demonstrated that the sham group had few TUNEL positive cells, which substantially increased following liver transplantation, consistent with the HE staining results. In contrast, salidroside pretreatment alleviated hepatic apoptosis in a dose-dependent manner (Fig. 2B,D).

### Salidroside regulates the TLR-4/NF-κB/NLRP3 inflammatory pathway in the liver of rats after liver transplantation

**Figure 3:**
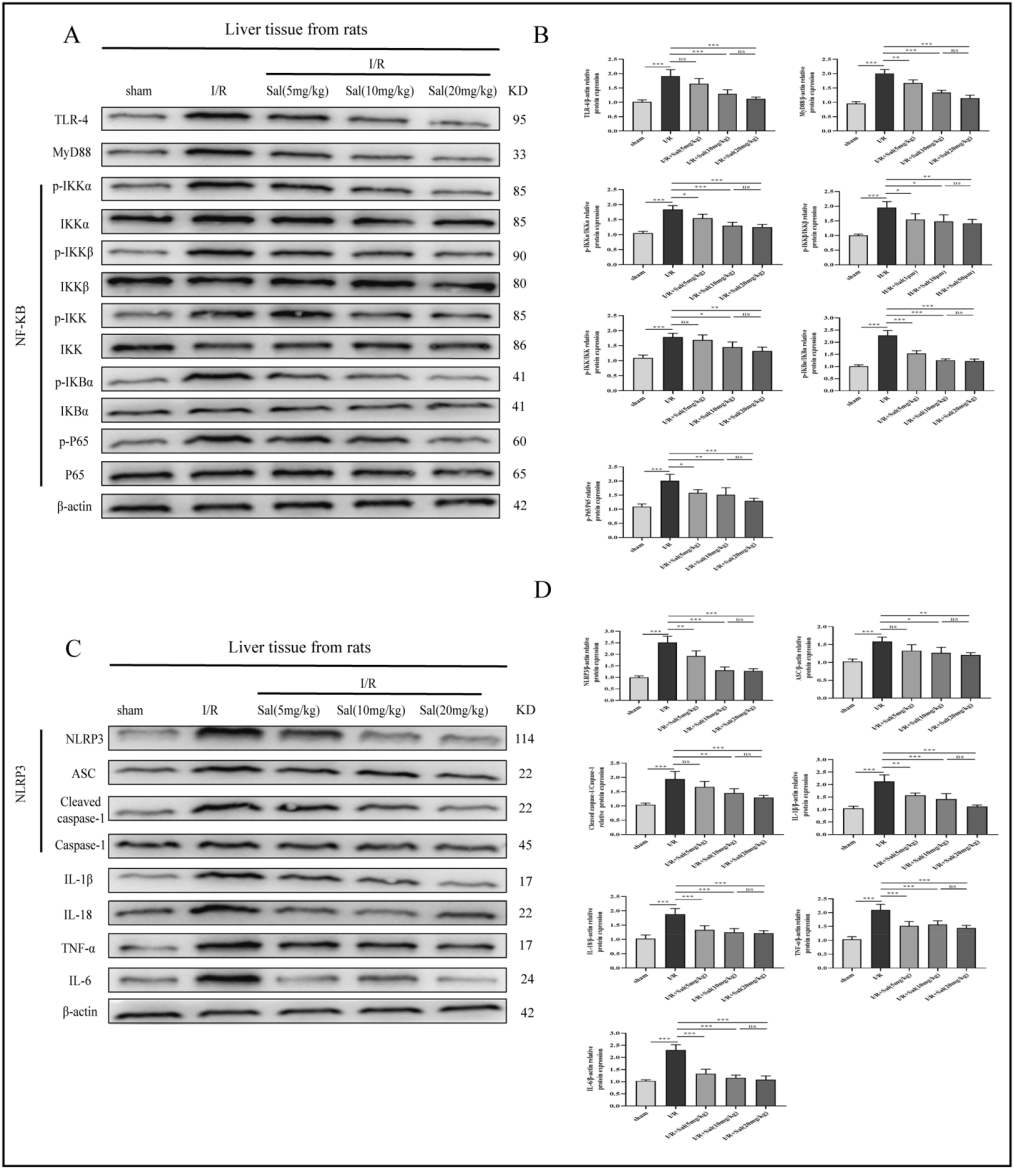
Salidroside inhibits TLR-4/NF-κB/NLRP3 inflammatory pathway in vivo. (**A**,**C**) Representative western blot assays showing TLR-4/NF-κB/NLRP3 inflammatory pathway associated proteins in the rat liver after liver transplantation. (**B**,**D**) Results of the Western blot experiments. The statistical differences among groups were assessed by one-way ANOVA. ^ns^*P* > 0.05, *P < 0.05, **P < 0.01, ***P < 0.001.

Liver tissues from I/R group exhibited significant increase in the expression of TLR-4, MyD88, p-IKKα, p-IKKβ, p-IKK, p-IκBα, p-P65, NLRP3, ASC, Cleaved caspase-1, IL-1β, IL-18, TNF-α and IL-6 proteins compared to those in the sham group. As expected, pretreatment with salidroside (5, 10, or 20 mg/kg) reduced activation of the TLR-4/ NF-κB /NLRP3 inflammatory pathway (Fig. 3).

### Salidroside mitigates hepatic IRI-induced inflammation and neutrophil infiltration

**Figure 4:**
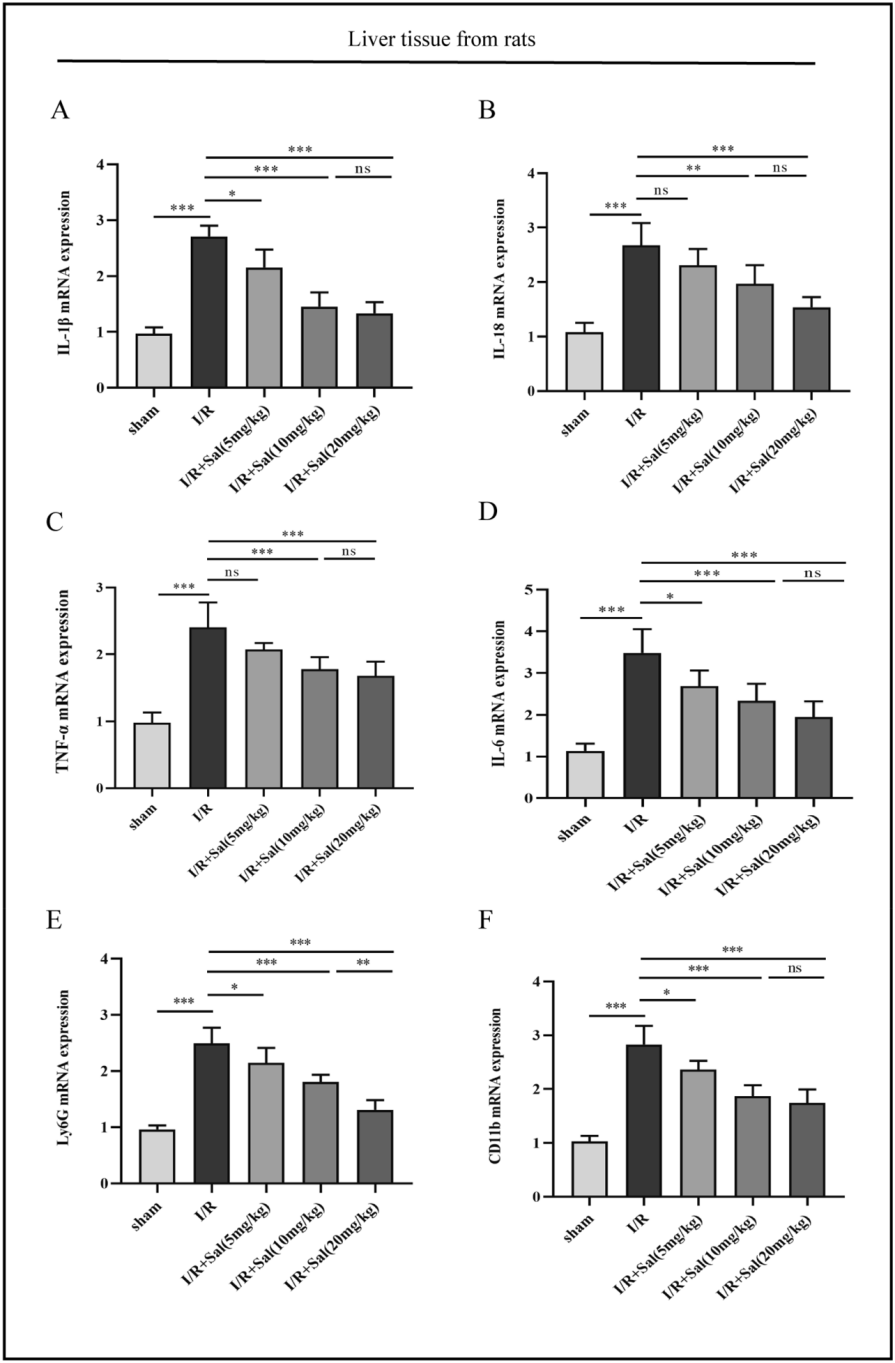
Salidroside suppresses transcription of pro-inflammatory cytokinesis. (**A**) IL-1β mRNA expression. (**B**) IL-18 mRNA expression. (**C**) TNF-α mRNA expression. (**D**) IL-6 mRNA expression. (**E**) Ly6G mRNA expression. (**F**) CD11b mRNA expression. The statistical differences among groups were assessed by one-way ANOVA. ^ns^*P* > 0.05, *P < 0.05, **P < 0.01, ***P < 0.001.

To establish a connection between neutrophil infiltration and liver inflammation, qRT-PCR was conducted to determine the expression levels of Pro-inflammatory cytokines and neutrophil markers, such as IL-1β, IL-18, IL-6, TNF-α, Ly6G and CD11b. Our analyses showed significantly elevated IL-1β, IL-18, IL-6 TNF-α, Ly6G and CD11b mRNA expression levels in the liver of the rats following liver transplantation, which were suppressed in the rats in the I/R + Sal (5, 10 or 20 mg/kg) group. The results of qRT-PCR indicated that salidroside pretreatment could mitigate hepatic IRI-induced inflammation and neutrophil infiltration in a dose-dependent manner (Fig. 4).

### Salidroside induced macrophage M2 polarization and alleviates the H/R-induced inflammation through the TLR-4/NF-κB/NLRP3 inflammatory pathway

**Figure 5:**
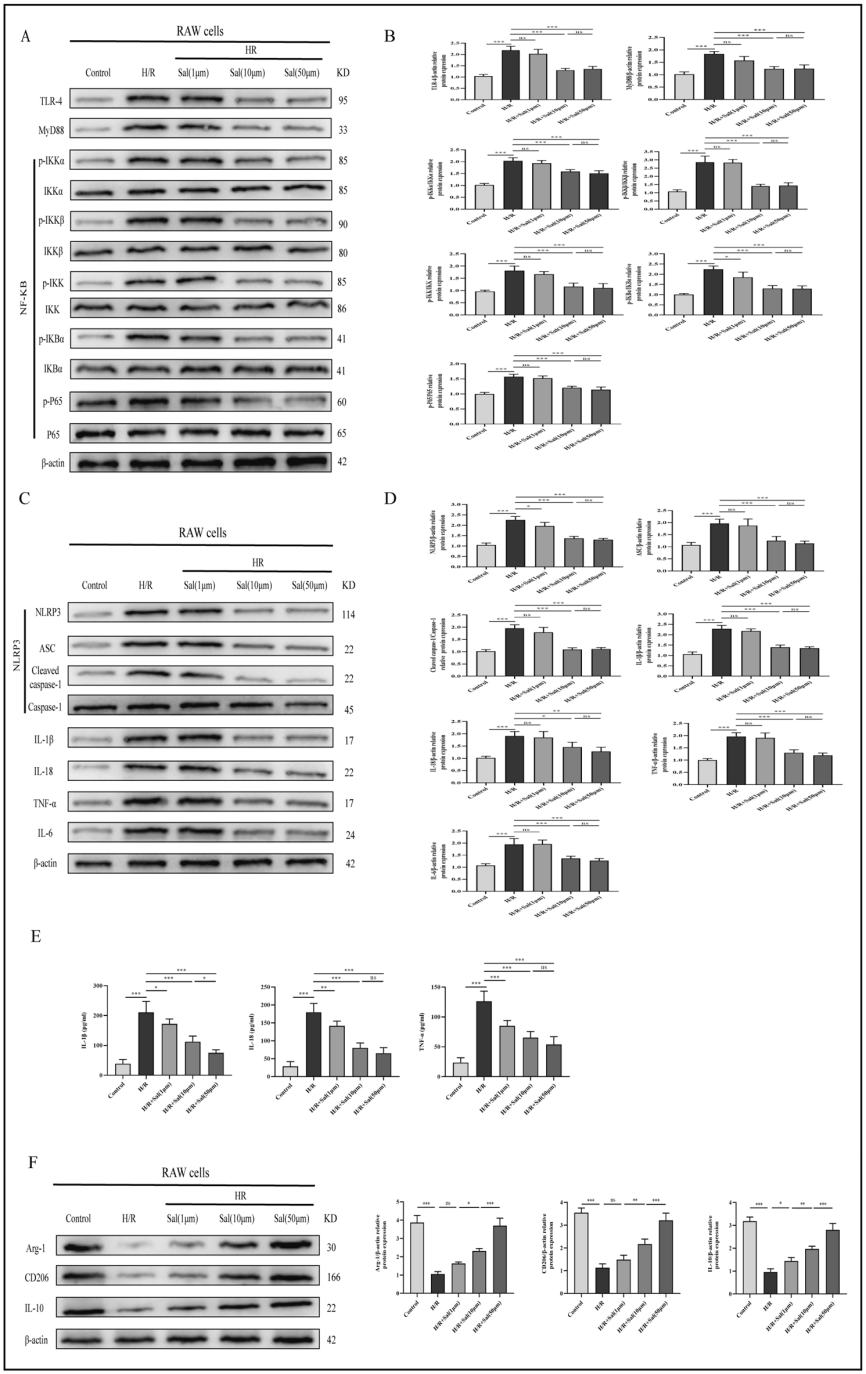
Salidroside inhibits inflammation in the H/R-exposed macrophages by regulating TLR-4/NF-κB/NLRP3 inflammatory pathway. (**A**,**C**) Representative Western blot data showing TLR-4/NF-κB/NLRP3 inflammatory pathway associated proteins in macrophages. (**B**,**D**) The results of western blot experiments. (**E**) Expression of IL-1β, IL-18 and TNF-α in macrophages was analyzed by ELISA. (**F**) Expression of M2 polarization markers in macrophages was analyzed by western blot. The statistical differences among groups were assessed by one-way ANOVA. ^ns^*P* > 0.05, *P < 0.05, **P < 0.01, ***P < 0.001.

Notably, our data showed that H/R exposure elevates the TLR-4, MyD88, p-IKKα, p-IKKβ, p-IKK, p-IκBα, p-P65, NLRP3, ASC, Cleaved caspase-1, IL-1β, IL-18, TNF-α and IL-6 protein levels compared to those in the control group. Treatment with salidroside (1 μM, 10 μM or 50 μM) diminished the activation of TLR-4/NF-κB/NLRP3 inflammatory pathway and decreased the secretion of the proinflammatory factors in a dose-dependent manner. Although there were significantly higher levels of IL-1β, IL-18 and TNF-α in the cell supernatant of the H/R group, treatment with salidroside (1 M, 10 M, or 50 M) decreased the levels of these cytokines in a dose-dependent manner. Western blot analysis was used to determine the expression levels of M2 polarization-related proteins (Arg-1, CD206, IL-10). As the salidroside concentration increased, the M2 polarization effect of macrophage gradually increased (Fig. 5).

### Protective effect of salidroside is attenuated after TLR-4 activation

**Figure 6:**
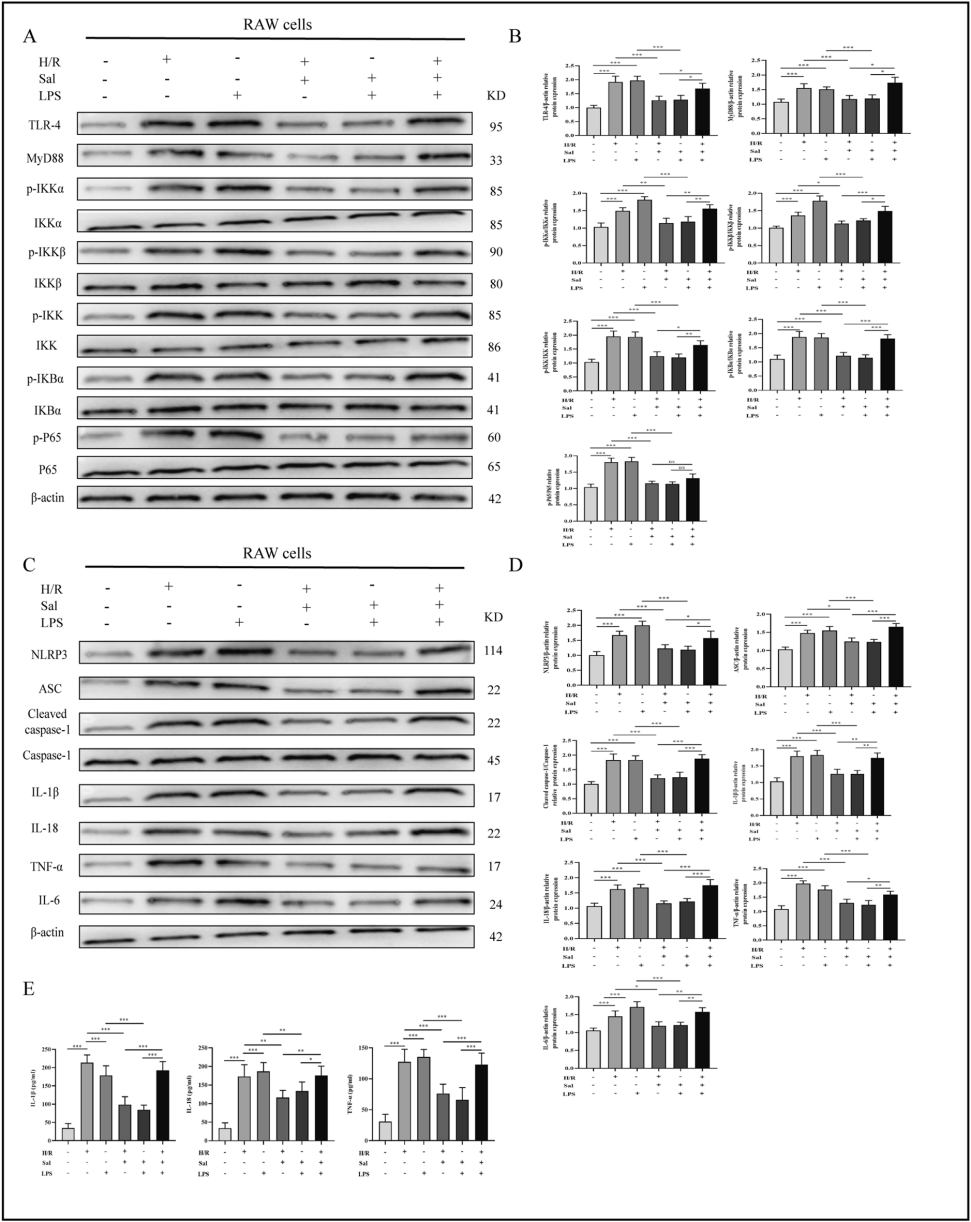
LPS (TLR-4 activator) partially alleviates the anti-inflammatory ability of salidroside via regulation of the TLR-4/NF-κB/NLRP3 inflammatory pathway. (**A**,**C**) Representative Western blot data of the TLR-4/NF-κB/NLRP3 inflammatory pathway associated proteins in macrophages. (**B**,**D**) The results of western blot experiments. (**E**) Production of IL-1β, IL-18 and TNF-α in macrophages was assessed by ELISA. The statistical differences among groups were assessed by one-way ANOVA. ^ns^*P* > 0.05, *P < 0.05, **P < 0.01, ***P < 0.001.

In comparison with the H/R + Sal (10 M) group, Western blotting analyses revealed that lipopolysaccharides (LPS) treatment significantly boosted the expression of TLR-4/NF-κB/NLRP3 inflammatory pathway related proteins (Fig. 6A–D). Similarly, compared to the H/R + Sal (10 μM) group, LPS significantly elevated the inflammatory cytokines in the cell supernatants (Fig. 6E). These data demonstrated that LPS (TLR-4 agonist) partly reversed the effects of salidroside. In addition, LPS stimulation significantly activated TLR-4/NF-κB/NLRP3 inflammatory pathway and facilitated the production of inflammatory cytokines by macrophages. Interestingly, the protective effects of salidroside in the macrophages with H/R exposure were attenuated after TLR-4 activation.

## Discussion

Hepatic IRI caused by liver transplantation is one of the main causes of impaired liver function^20^. Increased ischemia time and enhanced liver inflammatory responses may result in a poorer prognosis and overall survival^21^. Although the etiology of hepatic IRI is complex, previous studies have demonstrated the important role of excessive inflammatory response in donor liver, which is one of the important factors leading to compromised liver functions^22^. Thus, pharmaceutical approaches targeting proinflammatory cytokines could lay the basis for development of hepatic IRI therapy.

Salidroside, one of the most active chemicals from Rhodiola plants, has been associated with remarkable biological activities, including anti-inflammatory, antioxidant, and hepatoprotective effects^23–25^. For instance, Gao et al. showed that salidroside reduces cartilage degradation by modulating inflammation and immunological responses through the NF-κB pathway^26^. Besides, Zheng and Yang demonstrated that salidroside improved hepatic lipid metabolism and activation of the NLRP3 inflammasome, ultimately alleviating nonalcoholic fatty liver disease^27^. However, the mechanism underlying the therapeutic effects of salidroside in the rat hepatic IRI following liver transplantation has not been fully investigated. Herein, we verified that salidroside improved liver function inhibit hepatocyte apoptosis, decreased the release of inflammatory cytokines in vitro, similarly suppressing inflammatory responses in vitro. Moreover, our studies suggested that salidroside exerts its hepatoprotective effects by inhibiting the inflammatory response through regulating the TLR-4/NF-κB/NLRP3 inflammatory pathway. Our results may provide some novel insights into the therapy of hepatic IRI.

In addition, our study showed that treatment with salidroside (5, 10 or 20 mg/kg) significantly decreased serum AST and ALT levels in rats after liver transplantation. We also demonstrated that salidroside treatment alleviated hepatic histopathological changes and inhibited hepatocyte apoptosis in the liver tissues of rats following liver transplantation, indicating the activities of salidroside in IRI-induced liver injury. Our findings agreed with previous studies which showed that salidroside confers hepatoprotective properties^28^.

**Figure 7:**
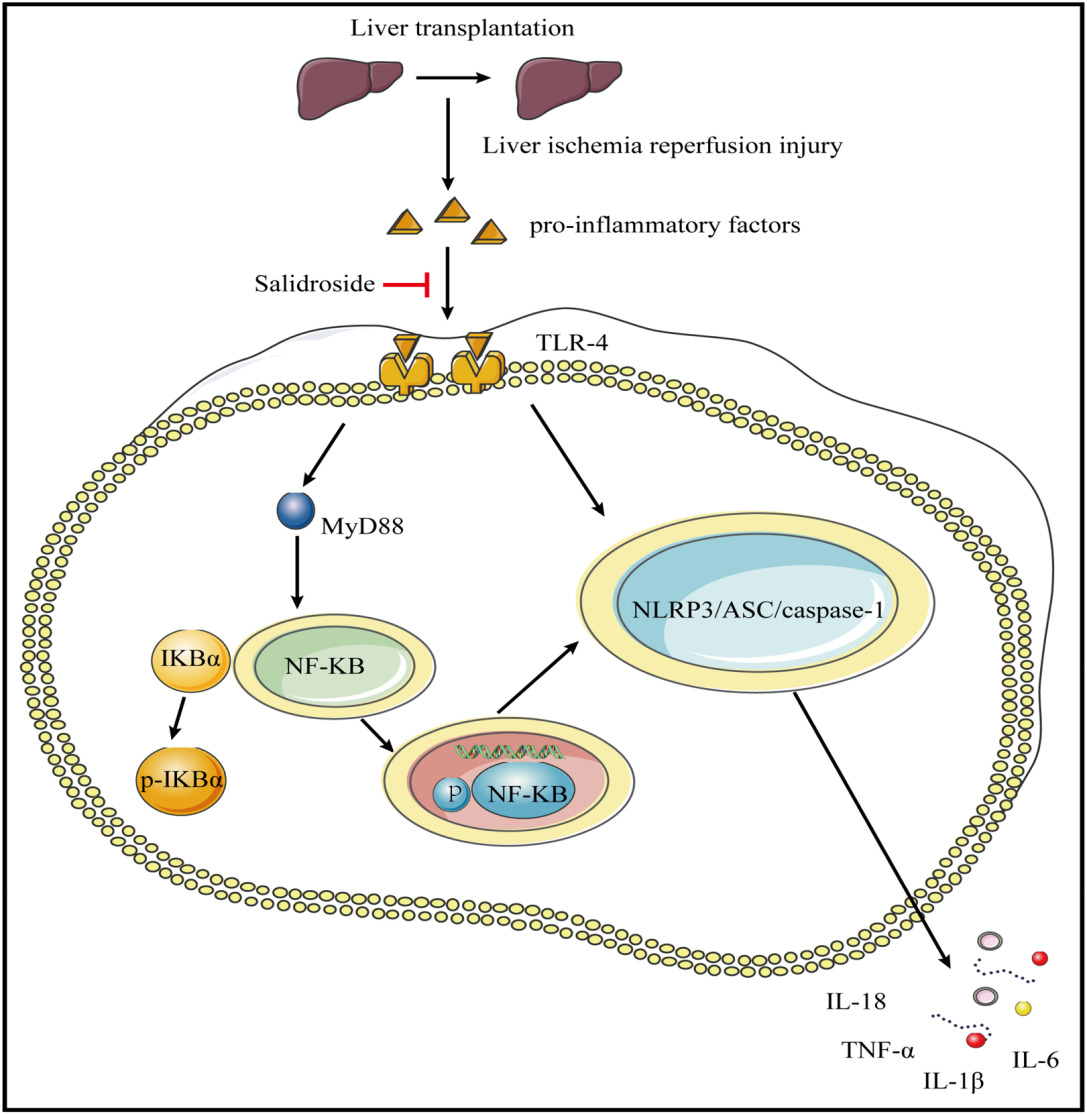
Putative route by which salidroside attenuates inflammatory reactions in liver IRI. The mechanisms involved in salidroside inhibition of hepatic IRI-induced inflammatory response through the roles of TLR-4/MyD88/NF-κB and TLR-4/NF-κB/NLRP3 inflammatory pathway.

Overproduction of inflammatory cytokines such as IL-1β, IL-18, IL-6, and TNF-α during the activation of IRI-induced immune responses is referred to as liver inflammation. Here, inflammatory substances accumulate in the liver and serum, aggravating endothelial cell damage and disrupting liver microcirculation through chemotaxis^29,30^. Our findings showed that salidroside alleviates hepatic IRI by suppressing the inflammatory responses. The inflammatory cytokines such as IL-1β, IL-18, IL-6, and TNF-α were shown to be overexpressed in both in vivo and in vitro hepatic IRI models. Furthermore, we showed that salidroside could inhibit the activation of the TLR-4/NF-B/NLRP3 inflammatory pathway in hepatic IRI, as well as the release of downstream inflammatory cytokines. The correlation between hepatic IRI and TLR-4/NF-κB signaling pathway has been reported. Hepatic IRI activates the TLR-4-mediated MyD88-dependent pathway and upregulates NF-κB which induces liver injury^8,31^. Besides, previous study has shown that damage-associated molecular pattern (DAMP) activates TLR-4 and MyD88, causing NF-κB to translocate to the nucleus via phosphorylation of the IκB at Ser32 and proteasome-mediated degradation^32^. The significance of TLR-4/NF-κB pathway in the activation of the NLRP3 inflammasome has already been reported^33^. Furthermore, NLRP3 promoted the recruitment of ASC, which then activated pro-caspase-1 into casepase-1, resulting in the production of mature IL-1β and IL-18, ultimately amplifying inflammatory responses and impairment^34^. The aberrant activation of the TLR-4/NF-κB/NLRP3 is quite significant in the formation and maintenance of the inflammatory microenvironment during hepatic IRI^35^. Our findings revealed that salidroside significantly reduces the expression of TLR-4, MyD88, p-IKKα, p-IKKβ, p-IKK, p-IκBα, p-P65, NLRP3, ASC, Cleaved caspase-1, IL-1β, IL-18, TNF-α and IL-6 in rats after liver transplantation as well as H/R-induced macrophages. Further analysis showed that salidroside reduces inflammation and hepatic IRI by inhibiting TLR-4-mediated NF-κB and inflammasome activation. Our data showed that TLR-4/NF-κB/NLRP3 inflammatory pathway was activated in rats after liver transplantation and in H/R induced macrophages. In addition, activation of the TLR-4/NF-κB/NLRP3 inflammatory pathway was inhibited by salidroside, and these effects were reversed by a TLR-4 agonist, suggesting that salidroside alleviate hepatic IRI by inhibiting the activation of TLR-4/NF-κB/NLRP3 inflammatory pathway and blocking TLR-4 (Fig. 7). Whereas our study highlights important findings, it is limited by the fact that we used a syngeneic liver transplantation mode to study liver transplantation IRI, which is not a perfect clinical model for studying allogeneic liver transplantation. Besides, we lacked clinical evidence to support the salidroside's effectiveness.

## Conclusions

In conclusion, our data illustrated that salidroside is an anti-inflammatory compound and alleviates hepatic IRI by inhibiting TLR-4/NF-κB/NLRP3 inflammatory pathway. Thus, salidroside could be a novel therapeutic strategy in the treatment of hepatic IRI.

## Supplementary Information

Supplementary Information 1.

Supplementary Information 2.

## Data Availability

All data generated or analyzed during this study are included in this published article and supplementary material S1.

## Abbreviations

ALT: Alanine aminotransferase
ASC: Apoptosis-associated speck-like protein
AST: Aspartate aminotransferase
DAPI: 4,6-Diamidino-2-phenylindole
MYD88: Myeloid differentiation factor 88
HE: Hematoxylin–eosin
H/R: Hypoxia/reoxygenation
IL-1β: Interleukin-1β
IL-6: Interleukin-6
IL-18: Interleukin-18
I/R: Ischemia/reperfusion
IRI: Ischemia/reperfusion injury
NF-κB: Nuclear factor kappa B
NLRP3: NLR family pyrin domain-containing 3
NLRs: Nucleotide-binding oligomerization domain-like receptors
PRRs: Pattern recognition receptors
SD: Sprague dawely
TLR-4: Toll-like receptor 4
TUNEL: Terminal deoxynucleotidyl transferase dUTP nick-end labeling
TNF-α: Tumor necrosis factor-α
