# Supplementary material for: Salidroside alleviates hepatic ischemia–reperfusion injury during liver transplant in rat through regulating TLR-4/NF-κB/NLRP3 inflammatory pathway
Source: Sci Rep. 2022 Aug 17;12:13973. doi: 10.1038/s41598-022-18369-4 (PMC9385636; doi:10.1038/s41598-022-18369-4)
Supplement: Supplementary file 2 — Supplementary Information 2. [file 41598_2022_18369_MOESM2_ESM.pdf]

Figure 3 NF-KB

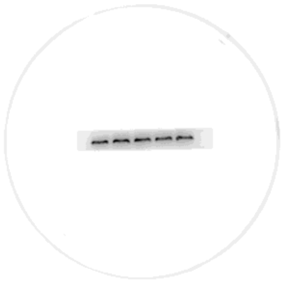

IKBα

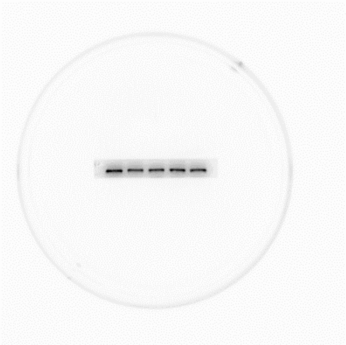

IKK

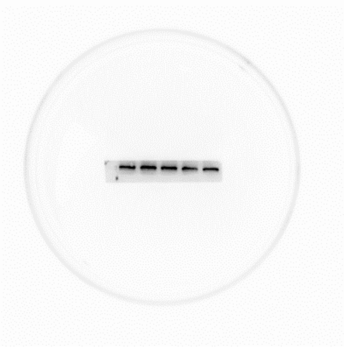

IKKα

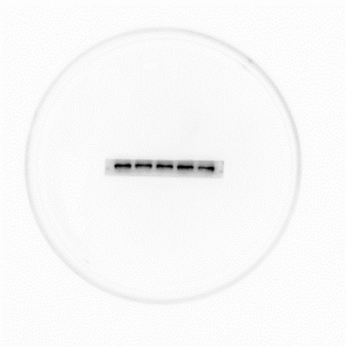

IKKβ

MyD88

P65

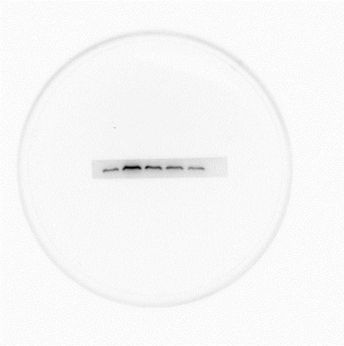

p-IKBα

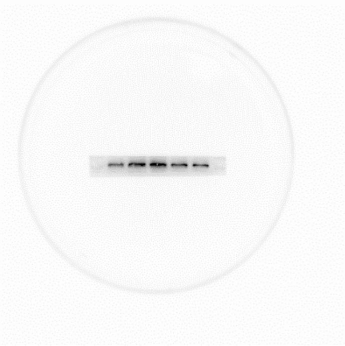

p-IKK

p-IKKα

p-IKKβ

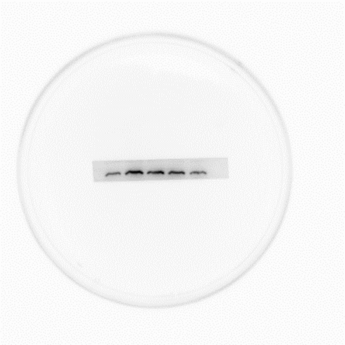

p-P65

TLR-4

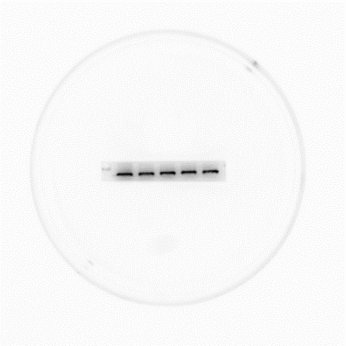

β -actin

Figure 3 NLRP3

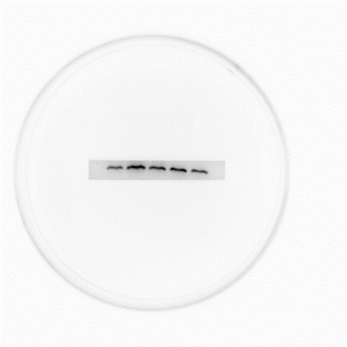

ASC

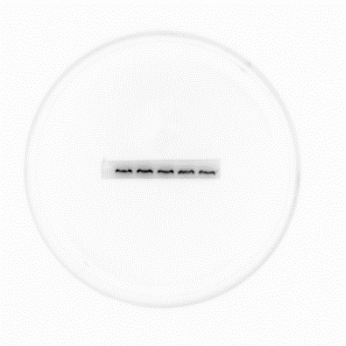

caspase-1

cleaved caspase-1

IL-1β

IL-6

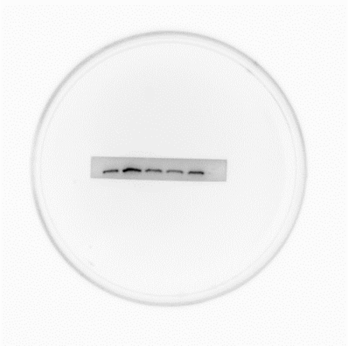

IL-18

NLRP-3

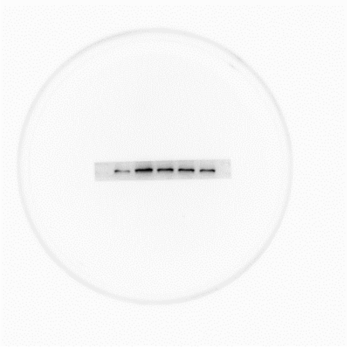

TNF-α

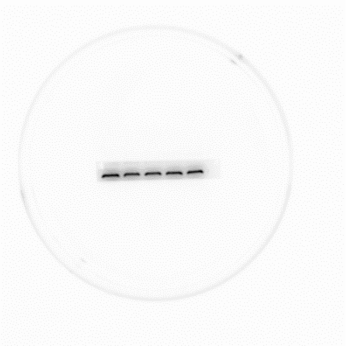

β -actin

Figure 5 NF-KB

IKBα

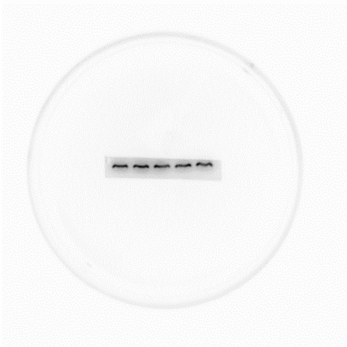

IKK

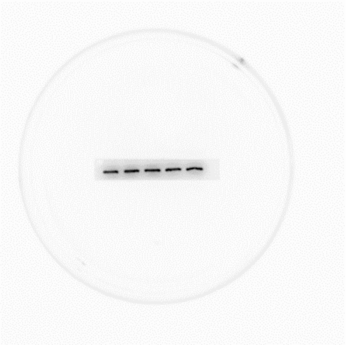

IKKα

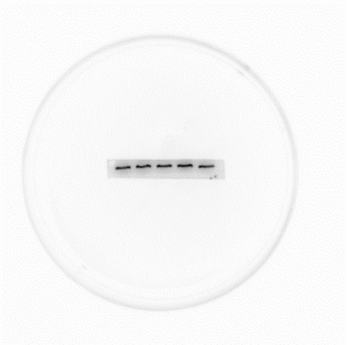

IKKβ

Myd88

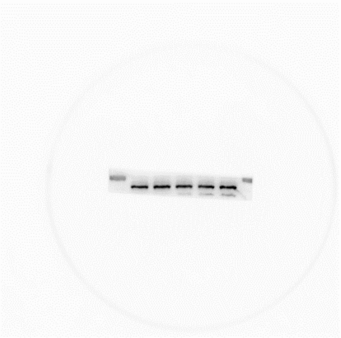

p65

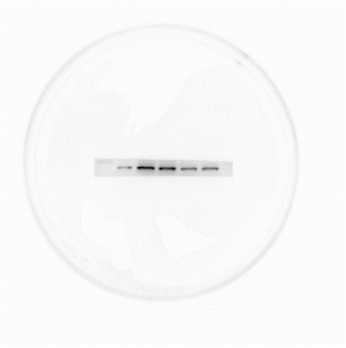

p-IKBα

p-IKK

p-IKKα

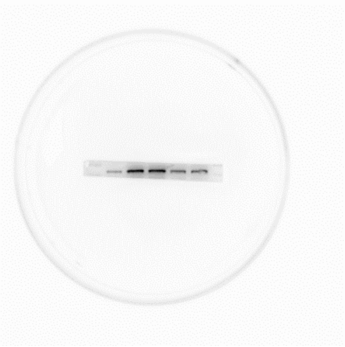
p-IKKβ

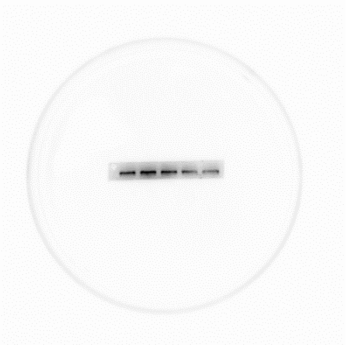
p-P65

TLR-4

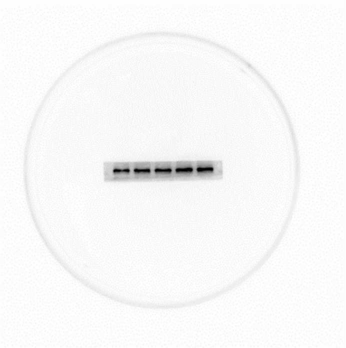
β -actin

Figure 5 NLRP3

ASC

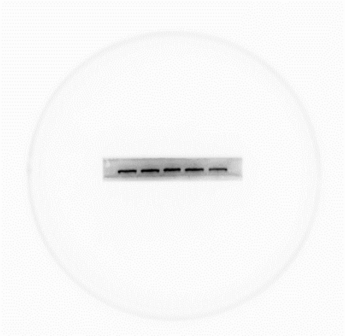
caspase-1

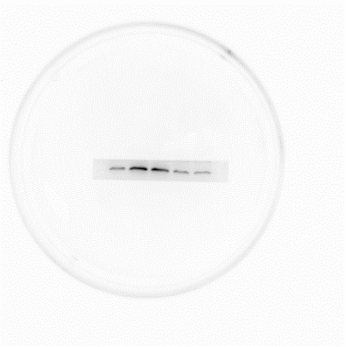

cleaved caspase-1

IL-1β

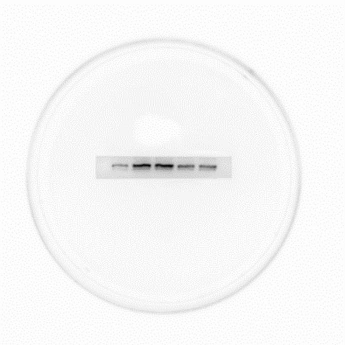

IL-6

IL-18

NLRP3

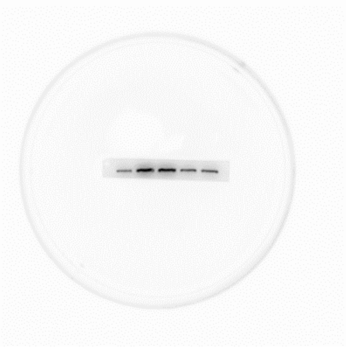

TNF-α

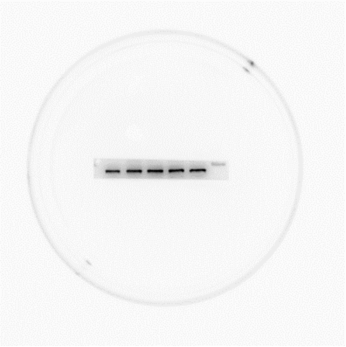

β -actin

Figure 6 NF-KB

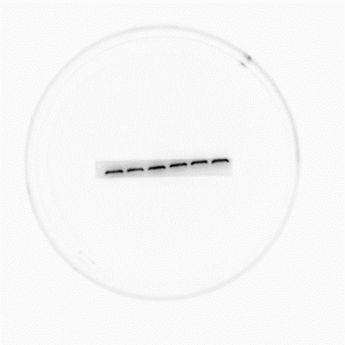

IKBα

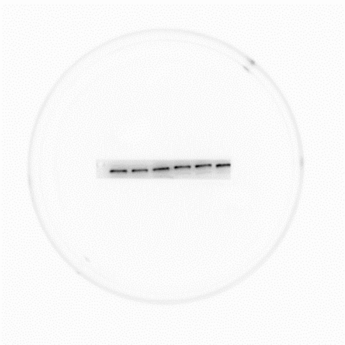

IKK

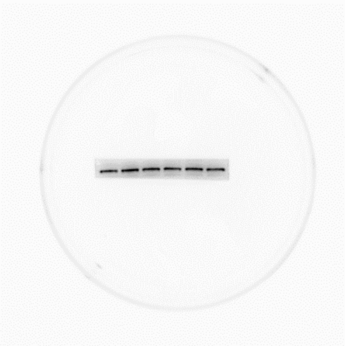

IKKα

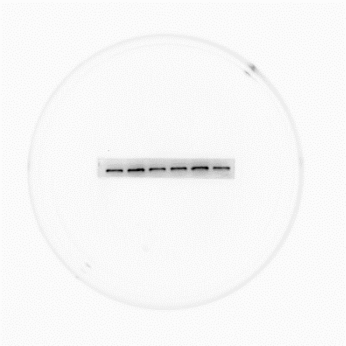

IKK-β

MyD88

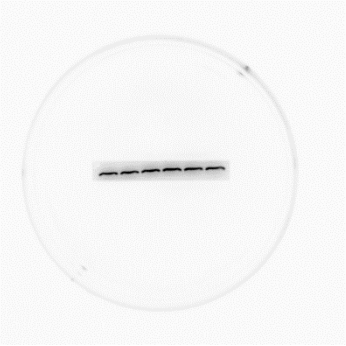

P65

p-IKBα

p-IKK

p-IKKα

p-IKKβ

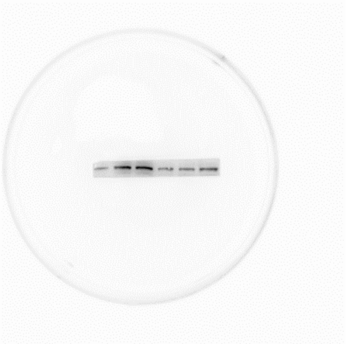

p-P65

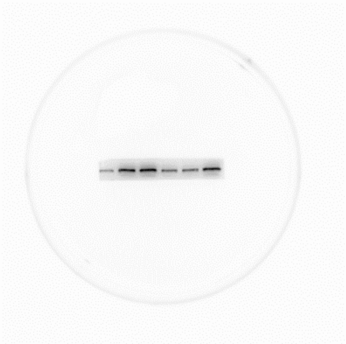

TLR-4

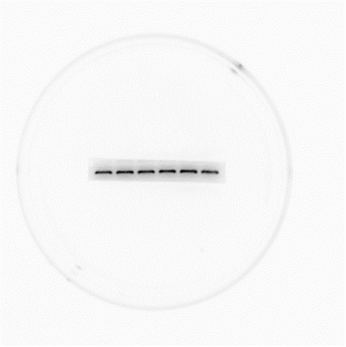

β -actin

Figure 6 NLRP3

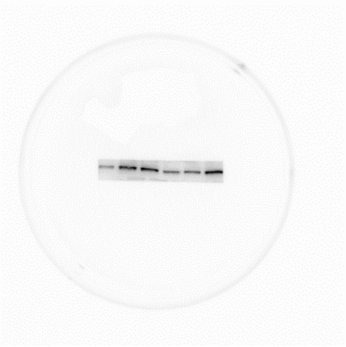

ASC

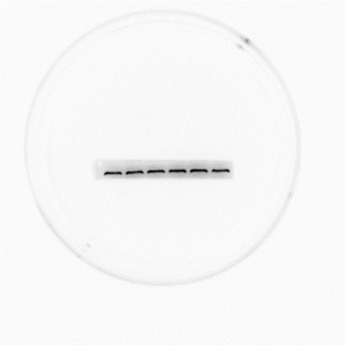

caspase-1

cleaved caspase-1

IL-1β

IL-6

IL-18

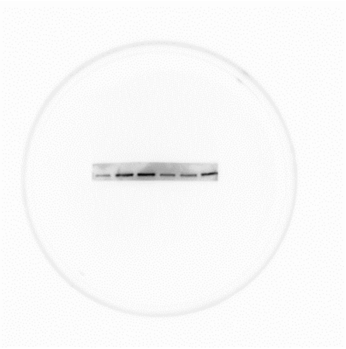

NLRP3

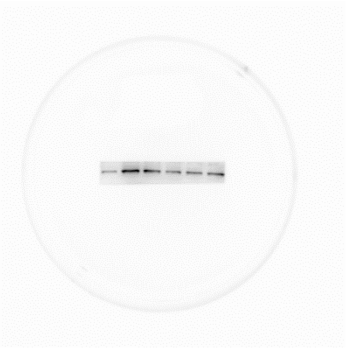

TNF-α

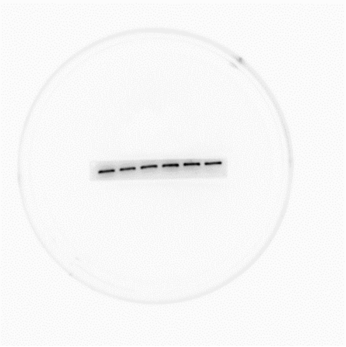

β -actin
